# Quinolone Resistance and Zoonotic Potential of *Corynebacterium ulcerans* from Domestic Animals in Brazil

**DOI:** 10.3390/antibiotics14080843

**Published:** 2025-08-20

**Authors:** Fernanda Diniz Prates, Max Roberto Batista Araújo, Jailan da Silva Sousa, Lincoln de Oliveira Sant’Anna, Tayná do Carmo Sant’Anna Cardoso, Amanda Couto Calazans Silva, Siomar de Castro Soares, Bruno Silva Andrade, Louisy Sanches dos Santos, Vasco Ariston de Carvalho Azevedo

**Affiliations:** 1 Operational Technical Nucleus, Microbiology, Hermes Pardini Institute, Vespasiano 33200-000, MG, Brazil; maxbarau@ufmg.br; 2 Institute of Biological Sciences, Federal University of Minas Gerais, Belo Horizonte 31270-901, MG, Brazil; jailanss@ufmg.br; 3 Laboratory of Diphtheria and Corynebacteria of Clinical Relevance, Department of Microbiology, Immunology and Parasitology, Rio de Janeiro State University, Rio de Janeiro 20550-013, RJ, Brazil; santanna.lincoln@posgraduacao.uerj.br (L.d.O.S.); santos.louisy@uerj.br (L.S.d.S.); 4 Institute of Biological and Natural Sciences, Federal University of Triângulo Mineiro, Uberaba 38025-350, MG, Brazil; siomar.soares@uftm.edu.br; 5 Laboratory of Bioinformatics and Computational Chemistry, Department of Biological Sciences, State University of Southwest of Bahia, Jequié 45083-900, BA, Brazil; bandrade@uesb.edu.br; 6 France’s National Research Institute for Agriculture, Food and Environment, 75338 Rennes, France

**Keywords:** *Corynebacterium ulcerans*, non-toxigenic, virulence factors, resistance genes, CRISPR–Cas system, quinolone resistance

## Abstract

Background: *Corynebacterium ulcerans* is an emerging zoonotic pathogen capable of cau-sing diphtheria-like infections in humans. Objectives: we report, for the first time in Brazil, the detection and phenotypic/genomic characterization of three atoxigenic ST-339 strains isolated from domestic animals, including one with a ciprofloxacin resistance profile linked to double GyrA mutations (S89L, D93G). Methods: species identification was performed by MALDI-TOF MS, followed by in vitro antimicrobial susceptibility testing, whole-genome sequencing, and bioinformatic analyses to predict virulence determinants, antimicrobial resistance genes, CRISPR–Cas systems, mobile genetic elements, and in silico structural analysis as well as phylogenetic reconstruction. Results: whole-genome sequencing confirmed species identity, revealed high genetic similarity, and identified distinct phylogenetic subclades, suggesting potential international dissemination. Genomic analyses showed conserved virulence determinants, such as incomplete pilus clusters, iron acquisition systems, and the *pld* gene, with the absence of the *tox* gene. Molecular modeling and dynamics simulations indicated that GyrA mutations disrupt critical ciprofloxacin–magnesium–water interactions, reducing binding stability. Mobile genetic elements, prophages, and CRISPR–Cas systems underscored the genomic plasticity of these isolates. Conclusions: these findings document a little-studied antimicrobial resistance mechanism in zoonotic *C. ulcerans*, highlighting the need for strengthened surveillance and further research on virulence and resistance, even in ato-xigenic strains.

## 1. Introduction

The genus *Corynebacterium* includes 166 valid species, some of them pathogenic to humans and animals [1]. *Corynebacterium diphtheriae*, the type species, is the leading cause of diphtheria, a potentially fatal disease characterized by local and systemic effects due to diphtheria toxin (DT), an exotoxin produced by the microorganism when lysogenized by phages carrying the *tox* gene [2,3]. Despite the global decline in cases following widespread vaccination, diphtheria remains endemic in some regions and is still reported among vaccinated individuals [4,5,6], with concerns heightened by declining vaccination coverage during the COVID-19 pandemic [7]. Consequently, the World Health Organization and the Pan American Health Organization (WHO/PAHO) have issued alerts emphasizing the need for preventive actions.

Besides *C. diphtheriae*, the closely related species *Corynebacterium belfantii*, *Coryneba-cterium pseudotuberculosis*, *Corynebacterium rouxii*, *Corynebacterium silvaticum*, and *Corynebacterium ulcerans*, which form the *C. diphtheriae* complex, may also carry the *tox* gene and produce the DT, causing diphtheria-like infections in humans and animals [8,9,10,11,12,13,14,15].

*C. ulcerans* is an emerging zoonotic pathogen that colonizes domestic [16,17] and wild animals [18,19], with human infections primarily linked to close contact with pets [20]. The number of toxigenic and atoxigenic *C. ulcerans* infections is increasing worldwide, as well as the severity of clinical symptoms, where toxigenic strains normally evolve into a diphtheria-like disease [21]. While early treatment with erythromycin or penicillin is recommended [22], resistance is emerging [4], prompting consideration of alternatives such as quinolones.

However, the limited genomic data constrain our understanding of *C. ulcerans* transmission and the emergence of toxigenic clones. A recent genomic study of 582 isolates from diverse hosts and regions applied a novel core genome genotyping approach, revealing major sublineages and diverse mobile elements, including five *tox* prophage families and a novel *tox*-carrying element, indicating interspecies prophage transfer within the *C. diphtheriae* complex [23].

Epidemiological data on potentially toxigenic *Corynebacterium* species from animals remain limited due to the lack of mandatory reporting in most countries, hindering public health efforts and our understanding of zoonotic transmission. This study specifically aimed to phenotypically and molecularly characterize three atoxigenic *C. ulcerans* strains isolated from domestic animals in Brazil, emphasizing the identification of virulence factors and antimicrobial resistance genes, particularly mutations conferring quinolone resistance.

## 2. Results

### 2.1. Phenotypic Identification and Antimicrobial Susceptibility Profile

To evaluate the phenotypic characteristics and antimicrobial susceptibility of the isolates, we performed colony morphology assessment, Gram staining, MALDI-TOF MS (Matrix-Assisted Laser Desorption Ionization Time-of-Flight) identification, and disk diffusion antimicrobial susceptibility testing according to the Brazilian Committee on Antimicrobial Susceptibility Testing (BrCAST) guidelines.

After 48 h of incubation, the colonies exhibited a dry, white, and opaque morphology on the surface of the blood agar. The Gram stain from these colonies revealed Gram-positive bacillary forms arranged in pallid shapes with angular formations between cells. In all cultures, MALDI-TOF MS analysis identified isolates as *C. ulcerans* (99% probability), which were named *C. ulcerans* IHP37393, IHP103889, and IHP106492. In the first culture (IHP37393), for ear injury in a dog from the State of Paraná, another colony was also identified as *Staphylococcus aureus* (99% probability). In the second culture (IHP103889), for neck abscess in a cat from the State of Pernambuco, *Enterococcus* spp. was also identified with 99% probability. Finally, in the third culture (IHP106492), for ear injury in a dog from the State of São Paulo, *Morganella morganii* was also identified with 99% probability. 

According to the halos in the disk diffusion method, the clinical isolates were categorized as sensitive to the following antimicrobials tested: clindamycin (IHP106492 = 15 mm; cut-off ≥ 15 mm), erythromycin (IHP37393 = 38 mm; IHP103889 = 38 mm; IHP106492 = 34 mm; cut-off ≥ 24 mm), linezolid (IHP37393 = 38 mm; IHP103889 = 36 mm; IHP106492 = 32 mm; cut-off ≥ 25 mm), rifampin (IHP37393 = 36 mm; IHP103889 = 36 mm; IHP106492 = 36 mm; cut-off ≥ 24 mm), trimethoprim-sulfamethoxazole (IHP37393 = 31 mm; IHP103889 = 35 mm; IHP106492 = 33 mm; cut-off ≥ 23 mm), and tetracycline (IHP37393 = 33 mm; IHP103889 = 36 mm; IHP106492 = 35 mm; cut-off ≥ 24 mm). Susceptibility with increased exposure was observed for benzylpenicillin (IHP37393 = 24 mm; IHP103889 = 20 mm; IHP106492 = 19 mm; cut-off 12–49 mm) and ciprofloxacin (IHP103889 = 35 mm; IHP106492 = 36 mm; cut-off 24–49 mm). Resistance to clindamycin (IHP37393 = 14 mm; IHP103889 = 14 mm; cut-off < 15 mm) and ciprofloxacin (IHP37393 = 0 mm; cut-off < 24 mm; confirmed by E-test > 32 µg/mL; cut-off > 0.5 µg/mL) was also observed.

### 2.2. Genome Features and Taxonomy

Whole-genome sequencing (WGS) was performed on all isolates, followed by genome assembly, annotation, and taxonomic classification using genome-based comparative tools to determine their genomic features and species identity.

The average genome size is approximately 2.5 Mb. The genomes had predicted guanine and cytosine (GC) contents of 53.3%. Relevant information about the assemblies of the IHP37393, IHP103889, and IHP106492 strains, as well as the median coverage, N50, number of CDSs, rRNA, and tRNA, is shown in Table 1. No chimerism was found.

All strains were classified as *C. ulcerans* by the Type Strain Genome Server (TYGS) [24] and Genome Taxonomy Database Toolkit (GTDB-Tk) v.2.4.0 [25]. The Average Nucleotide Identity (ANI) values obtained from the comparison between our strains and close reference genomes identified by TYGS are shown in a heatmap (Supplementary Materials Figure S1). The results showed ANI values of 99.9% for our strains in comparison to *C. ulcerans* NCTC 7910^T^. Moreover, as expected, the results of DNA–DNA hybridization (DDH) in silico comparing our genomes with the *C. ulcerans* NCTC 7910^T^ genome revealed values of 88.5%, 88.1%, and 88.4% for IHP37393, IHP103889, and IHP106492, respectively (Supplementary Materials Table S1).

### 2.3. Multilocus Sequence Typing (MLST) Characterization and Phylogenetic Analysis

MLST was conducted based on the sequences of seven housekeeping genes, and phylogenetic relationships among isolates were inferred from the single-nucleotide polymorphism (SNP) of the core genome.

Analysis of the housekeeping genes showed that all strains included in this study belong to ST-339. The allelic profile found was 41 (*atp*A), 35 (*dna*E), 79 (*dna*K), 49 (*fus*A), 52 (*leu*A), 48 (*odh*A), and 39 (*rpo*B).

**Figure 1:**
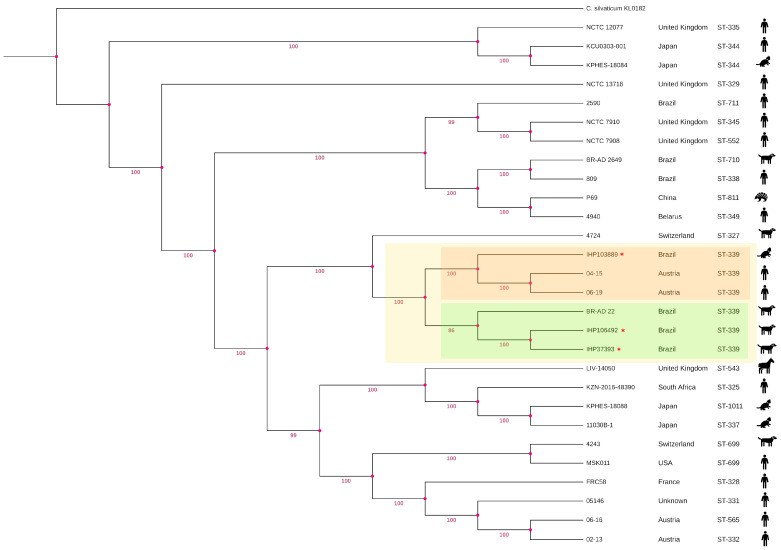
Phylogenetic tree based on single-nucleotide polymorphism of the core genome. The tree was built using IQ-TREE2 v.2.0.7, and the distance was obtained using the maximum likelihood method. Bootstrap values were calculated using 1000 replicates. The main cluster containing our strains is highlighted in a yellow square, while the subclusters are highlighted in orange and green squares.

In the phylogenetic analysis, it was possible to observe that all strains belonging to ST-339 formed a well-defined clade with strong bootstrap support (100%), highlighted in Figure 1 by a yellow square. This clade is divided into two subclades, which are highlighted in Figure 1 by orange and green squares.

The first subclade contained one of our strains (IHP103889) and two Austrian strains (04-15 and 06-19). The latter had more phylogenetic proximity to each other than to IHP103889, which was evidenced by another distinct subclade showing good bootstrap support (100%) between them. This is probably because these strains were isolated from humans, while IHP103889 was isolated from a cat. The other subclade was composed of two strains from this study (IHP37393 and IHP106492) and another Brazilian strain (BR-AD 22), all of which were isolated from dogs. Moreover, it was possible to observe that the ST-339 clade was separated from the *C. ulcerans* 4724 strain (ST-327) and isolated from a dog in Switzerland through a distinct clade showing a common ancestor among them and strong bootstrap support (100%).

### 2.4. Prediction of Mobile Genetic Elements and CRISPR–Cas Systems

Genomic analyses were carried out to identify plasmids, integrons, insertion sequences (ISs), prophages, CRISPRs-Cas systems using specialized bioinformatics tools to assess the genomic plasticity of the isolates.

No plasmids were detected in the genomes of our strains using PlasmidFinder v.2.1.6 [26]. In addition, IntegronFinder v.2.0 [27] did not identify any integrons in the genomes. Detailed analysis of the ISs annotated by ISEScan v.1.7.2.3 [28] identified three different IS families (IS110, IS21, IS256) and the same number of insertion sequences in the genome of each strain. In all of them, IS110 (2 in each strain) was the most abundant IS, followed by IS21 and IS256 (1 in each strain). The copy number of the annotated ISs was stable in all strains. All information about the IS copies in each strain, besides the transposase gene, is provided in Supplementary Materials Table S2.

**Figure 2:**
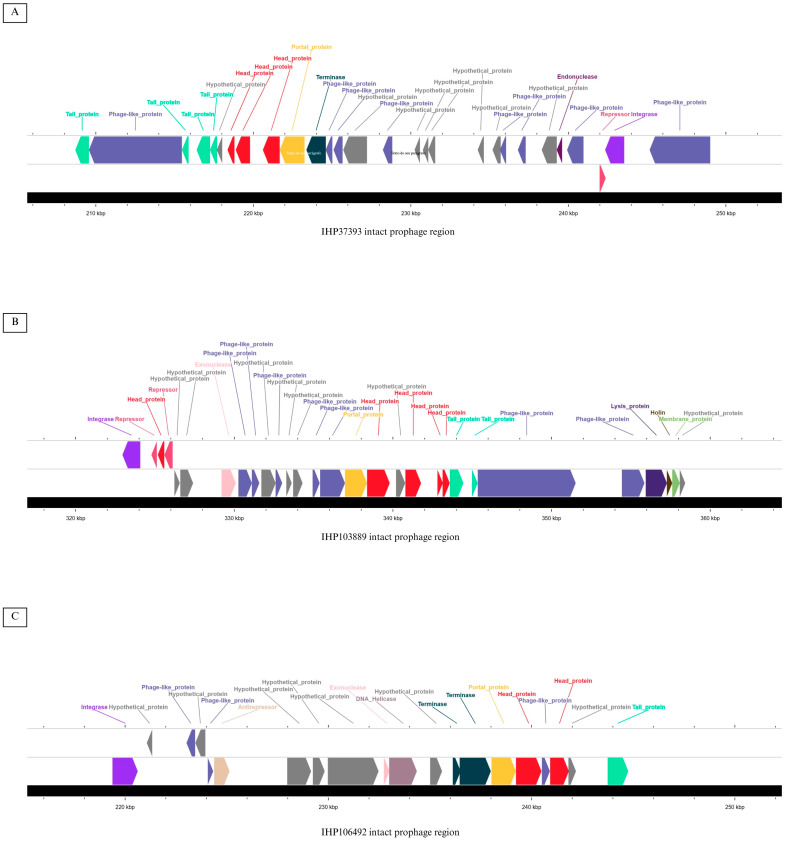
Linear genome map visualized using Circular Genome View Server in Proksee, showing the location of the intact prophage regions and predicted phage genes in the IHP37393 (**A**), IHP103889 (**B**), and IHP106492 (**C**) strains.

Using the Phage Search Tool with Enhanced Sequence Translation (PHASTEST) [29], we predicted only one intact prophage in the genome of the IHP37389 strain (region length: 47.9 Kb; completeness: 130; total number of CDSs: 28; region position: 201090-249006; most common phage: *Rhodococcus* phage Jace; GenBank accession number: NC047974; GC content: 55.58%). In the genome of the IHP103889 strain, two prophages were predicted, one intact prophage (region length: 38.2 Kb; completeness: 130; total number of CDSs: 29; region position: 322879-361082; most common phage: *Gordonia* phage Nyceirae; GenBank accession number: NC031004; GC content: 55.29%) and one questionable prophage (region length: 14.3 Kb; completeness: 80; total number of CDSs: 14; region position: 61225-75524; most common phage: *Gordonia* phage GMA5; GenBank accession number: NC030907; GC content: 57.32%). Lastly, in the genome of the IHP106492 strain, two prophages were also detected, one intact prophage (region length: 42.5 Kb; completeness: 100; total number of CDSs: 20; region position: 205082-247623; most common phage: *Corynebacterium* phage Adelaide; GenBank accession number: NC048791; GC content: 54.21%) and one questionable prophage (region length: 14.1 Kb; completeness: 90; total number of CDSs: 13; region position: 59635-73774; most common phage: *Gordonia* phage GMA5; GenBank accession number: NC030907; GC content: 55.11%). All phage genes predicted in the intact prophages of each strain are shown in Figure 2.

CRISPRCasFinder v.4.2.30 [30] identified a type I-E CRISPR–Cas system, lacking the *cse*1 gene in all strains. The IHP106492 strain harbored an additional type IU CRISPR–Cas system, which contained *cas*2, *cas*1, *cas*3, *csb*2, and *csb*1 genes. Three CRISPR arrays with evidence levels equal to 4 were found in each of the strains. Through an analysis of the spacer diversity among all CRISPR arrays, we found a total of 192 spacer sequences, and the IHP103889 strain carried the largest number of them, 82 spacers, followed by IHP37393 and IHP106492, with 55 spacers each. The CRISPRTarget database [31] identified 86 spacers: 24 spacers in IHP37393, 37 spacers in IHP103889, and 25 spacers in IHP106492. The identification of the spacers with the highest match values is shown in Supplementary Materials Table S3. In addition, linear genome maps including the location of the *cas* genes and some CRISPR arrays are shown in Supplementary Materials Figure S2.

### 2.5. Identification of Genes Encoding Antimicrobial Resistance and Virulence Factors

Genomes were screened against reference databases to predict antimicrobial resistance genes and virulence-associated determinants, enabling comparison with refe-rence *C. ulcerans* strains.

**Figure 3:**
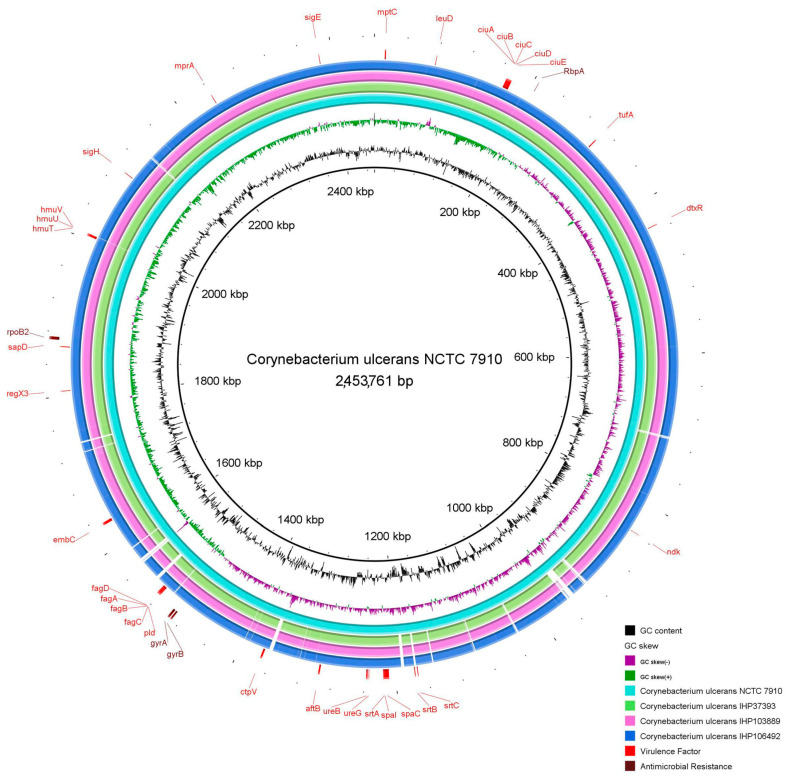
Circular comparative map of all complete genomes of *C. ulcerans* using BRIG software v.0.95. As a reference genome, we used *C. ulcerans* NCTC 7910^T^, which is represented in this map in the central position with the first three rings showing its size, GC content, and GC skew. Each outer ring represents the complete genome of one specific strain of *C. ulcerans*.

As shown in Figure 3, compared to the reference genome *C. ulcerans* NCTC 7910^T^, the incomplete pilus clusters *spa*ABC (*srt*A and *spa*C), *spa*DEF (*srt*B and *srt*C), and *spa*GHI (*spa*I) were predicted in all strains, encoding SpaA-, SpaD-, and SpaH-type pili, respectively. Moreover, all strains harbored one gene that encodes surface-anchored pilus proteins (*sap*D). Genes involved in the ABC transporter (*fag*ABC operon and *fag*D gene), the ABC-type heme transporter (*hmu*TUV cluster), Ciu iron uptake, the siderophore biosynthesis system (*ciu*ABCDE cluster), and the iron-dependent regulator of diphtheria toxin production (*dtx*R) were also identified in all strains.

The *emb*C, *mpt*C, and *aft*B genes, associated with the biosynthesis of lipoarabinomannan-like lipoglycan (CdiLAM), were also found in all isolates, as well as some urease- encoding genes, *ure*B and *ure*G, and the *ctp*V gene, a putative copper exporter. As expected, the *pld* gene encoding sphingomyelin-degrading phospholipase D was found in all strains. Other genes were predicted by PanViTa, including *leu*D, *mpr*A, *ndk*, *reg*X3, *sig*E, *sig*H, and *tuf*A; however, they presented a low identity percentage. The *tox* gene was not found in any of our strains. Genes involved in antimicrobial resistance, including *rpo*B2, *rbp*A, *gyr*A, and *gyr*B, were predicted in all strains.

### 2.6. Mutation Analysis

To investigate the genetic basis of ciprofloxacin resistance, we analyzed the quinolone resistance-determining regions (QRDRs) of *gyr*A and *gyr*B, followed by structural modeling, molecular docking, and molecular dynamics simulations to assess the impact of the detected mutations.

**Figure 4:**
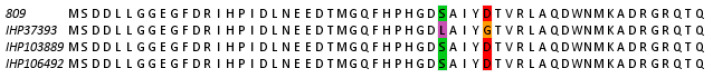
Multiple sequence alignment of the QRDRs of *Corynebacterium ulcerans* DNA gyrase subunit A, highlighting mutations (purple and orange colors) present in the ciprofloxacin-resistant IHP37393 strain.

DNA gyrase subunit A (GyrA) of the ciprofloxacin-resistant strain IHP37393 exhi-bited two mutations compared to the susceptible strains (Figure 4; Supplementary Materials Figure S3). The mutant protein has a leucine and a glycine at positions 89 and 93, respectively, whereas susceptible strains possess a serine and an aspartate at these positions [32]. In contrast, the DNA gyrase subunit B (GyrB) of the resistant strain showed no mutations relative to the susceptible strains.

The gyr89L-93G model, after undergoing a 5000-step energy minimization process using the steepest descent algorithm, exhibited 95.2% of amino acid residues in favored regions, while the remaining 4.8% were present in allowed regions (Supplementary Materials Figure S4A). Additionally, a 100-nanosecond simulation of the model in its apo conformation confirmed that the protein maintained a stable structure (Supplementary Figure S4B,C). These results suggest that the model is structurally stable and functionally viable, making it suitable for subsequent silico analyses.

**Figure 5:**
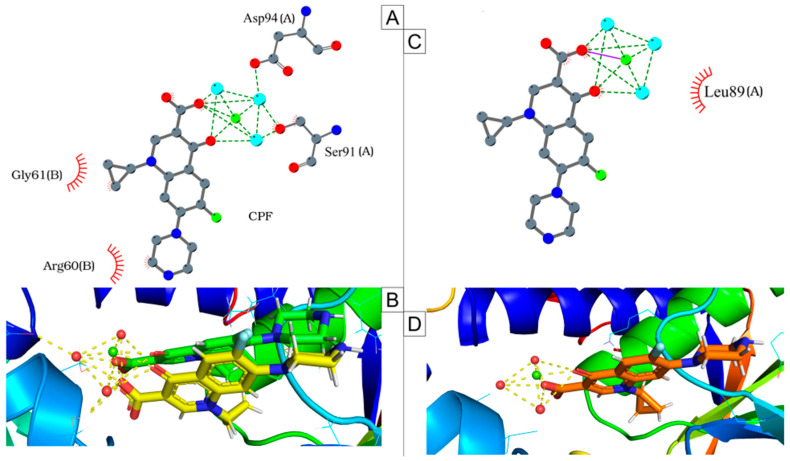
Docking simulations. (**A**,**B**) Redocking of the 5BTC—ciprofloxacin (CPF) complex (*M. tuberculosis* DNA gyrase complexed with ciprofloxacin), showing CPF’s interactions with magnesium ions (green spheres) and water molecules (blue and red spheres in (**A**) and (**B**), respectively). (**B**) Comparison of CPF’s position, with the original position in yellow and the redocked position in green. (**C**,**D**) CPF’s interactions with the mutated DNA gyrase (gyr89L-93G).

Molecular docking analysis revealed that the 5BTC–ciprofloxacin complex (*Mycoba-cterium tuberculosis* DNA gyrase complexed with ciprofloxacin) had a binding score of −6.204, which makes it comparable to the gyr89L-93G—ciprofloxacin complex, with a score of −6.246. However, in the 5BTC- ciprofloxacin complex, ciprofloxacin interacts with Ser91 and Asp94 from chain A via a water/magnesium ion bridge and undergoes hydrophobic interactions with arginine and glycine at positions 60 and 61 of chain B (Figure 5A,B). In contrast, in the gyr89L-93G—ciprofloxacin complex, the interaction at position 89 is wea-kened due to the serine-to-leucine substitution, resulting in a hydrophobic interaction in the region (Figure 5C,D). Additionally, the aspartate-to-glycine substitution at position 93 leads to a loss of interaction between the mutated protein and the water/magnesium ion bridge complex. It is worth noting that serine 91 and aspartate 94 in the 5BTC protein occupies the same positions as serine 89 and aspartate 93 in the gyr89L-93G model. Thus, despite the similar binding affinity energies obtained from molecular docking, our fin-dings indicate a significant loss of key interactions in the ciprofloxacin-bound mutated DNA gyrase, which impacts its binding stability and function.

**Figure 6:**
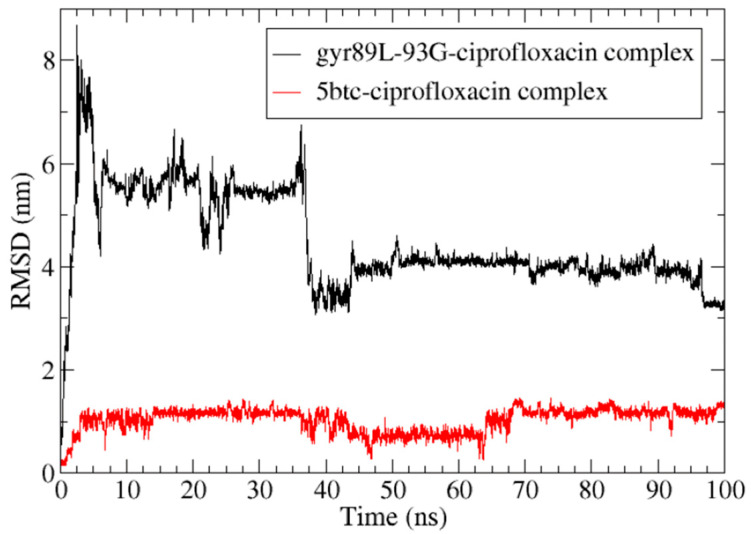
Root mean square deviation (RMSD) of ciprofloxacin following least-squares fitting to the complex backbone over a 100-nanosecond simulation.

The 100-nanosecond molecular dynamics simulation of the 5BTC—ciprofloxacin complex demonstrated that ciprofloxacin remained bound to the DNA gyrase protein throughout the entire simulation (Figure 6).

**Figure 7:**
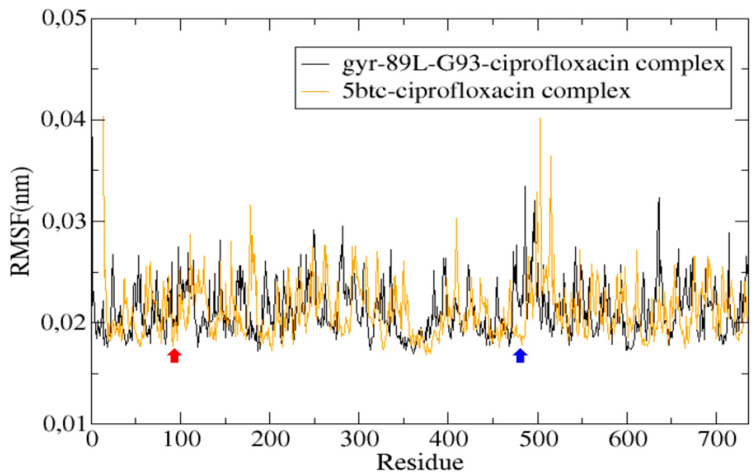
Root mean square fluctuation (RMSF) of the protein, illustrating amino acid movement throughout a 100-nanosecond simulation. The red arrow highlights amino acids in the QRDR of the GyrA subunit, while the blue arrow points to amino acids in the GyrB subunit that interact with ciprofloxacin.

Like other ciprofloxacin-susceptible *C. ulcerans* strains, the 5BTC DNA gyrase contains serine and aspartate at positions 89 and 93 in the GyrA subunit, highlighting these residues as key determinants of ciprofloxacin’s inhibitory activity (Figure 7).

**Figure 8:**
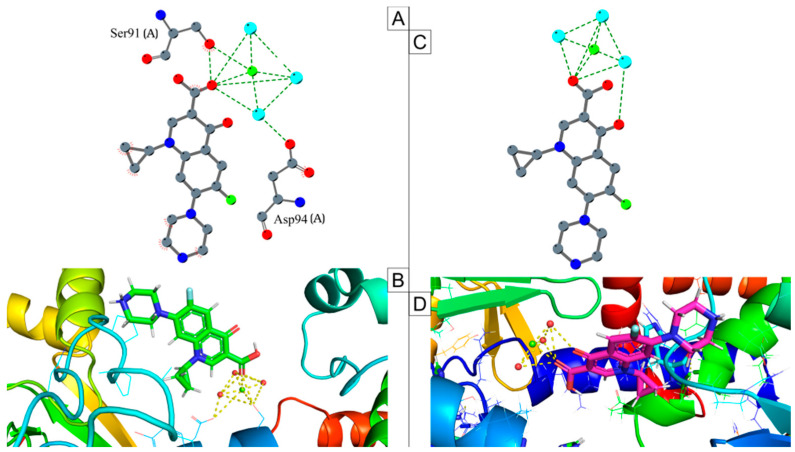
Protein–ligand interactions during molecular dynamics simulations. (**A**,**B**) Last frame of the 5BTC–ciprofloxacin complex simulation. (**C**,**D**) Fifth frame (0.04 nanoseconds) of the gyr89L-93G—ciprofloxacin complex simulation.

In contrast, the molecular simulation of the gyr89L-93G—ciprofloxacin complex revealed a highly unstable interaction, with the ligand dissociating from the enzyme’s active site within the first 10 nanoseconds of the simulation (Figure 6). The final frame of the 5BTC—ciprofloxacin simulation (Figure 8A,B) showed that, despite some positional fluctuations, ciprofloxacin-maintained interactions with the key amino acid residues throughout the simulation. Conversely, in the gyr89L-93G—ciprofloxacin complex, ciprofloxacin completely lost its interaction with the mutated protein almost immediately, with no interaction observed beyond 0.04 nanoseconds (Figure 8C,D).

## 3. Discussion

In the present study, we characterized three *C. ulcerans* strains (IHP37393, IHP103889, IHP106492) isolated from domestic animals in Brazil. Identified by MALDI-TOF, all the strains showed ANI values above the limit proposed for species definition (95–96%) [33] and dDDH values above the recommended cut-off point of 70% [34], confirming their classification. Given the limited genomic data available for this species, we conducted molecular typing and phylogenetic analysis.

Classical MLST remains a valuable tool for epidemiological studies, supporting the surveillance of *C. diphtheriae* complex species and contributing to outbreak investigations [35]. In the present study, all isolates belonged to sequence type ST-339, a lineage previously reported in atoxigenic *C. ulcerans* strains isolated from Austrian patients with skin infections [36], in a nasal sample from an asymptomatic dog in Brazil [37], and in a dog with an ulcerative lesion in Italy [38]. Additionally, ST-339 strains have been identified in Canada, France, and Germany according to the Institut Pasteur MLST database.

The detection of ST-339 across such geographically distant regions and diverse hosts raises the hypothesis of the potential international dissemination of this lineage, possibly facilitated by animal–human–environment interactions. The repeated identification of ST-339 in both human and animal samples suggests that certain clones may possess adaptive advantages favoring their persistence and spread across different ecological niches. This finding underscores the importance of integrating molecular typing data with global surveillance systems to better understand transmission dynamics and the potential for the transboundary circulation of *C. ulcerans* strains.

In our study, the phylogenetic analysis using the core genome, in addition to showing that our strains grouped with those that had the same ST, also showed the formation of distinct subclades among them, characterizing genetic diversity among the isolates. Corro-borating our findings, genetic diversity among *C. ulcerans* species has already been observed in a previously published study, in which the formation of two distinct clades was evidenced [39]. Interestingly, it was also observed that regardless of the formation of the clades, the genetic variability within both clades allowed isolates from humans and animals to be distributed heterogeneously in this study, once again showing the tremendous genetic variation among the isolates, thus suggesting significant adaptive evolution across the different hosts. Our results also allow us to reaffirm, mainly from an epidemiological point of view, that *C. ulcerans* strains are widely geographically distributed, including the isolates that present significant genetic variations.

Horizontal gene transfer is key to bacterial genome evolution, promoting survival, adaptability, and the acquisition of virulence and antimicrobial resistance genes, with mobile genetic elements (MGEs) playing a central role [40]. Thus, we analyzed our bacterial genomes for MGEs, including plasmids, integrons, insertion sequences, and prophages.

Plasmids and integrons contribute to the development of antimicrobial resistance and to the spread of virulence genes, conferring selective advantages to bacteria [41,42]. None were detected in our strains, consistent with other *C. ulcerans* studies. In contrast, *C. diphtheriae* harbors plasmids such as pNG2 (with erythromycin resistance) and pLRPD (with multidrug resistance) [43,44], as well as integrons carrying resistance genes [45].

Insertion sequences, the smallest and most common MGEs, significantly influence bacterial genome structure, plasticity, and function [46,47]. They vary in copy number and can mobilize within genomes and through other MGEs like plasmids and phages [48]. We identified three IS families (IS110, IS21, IS256) with identical copy numbers in our strains, a finding previously reported in *C. silvaticum* [9]. These IS families were originally isolated from *Streptomyces coelicolor*, *Pseudomonas aeruginosa*, and *S. aureus*, respectively [49], and have been associated with antimicrobial resistance in *Corynebacterium striatum* [50] and *Corynebacterium jeikeium* [51]. Particularly, ISs belonging to the IS256 family have been described to flank the transposons Tn5432 and Tn5716, which carry resistance genes, in *C. striatum* [51]. The transposon Tn5432 has also been detected in other corynebacterial species, including *C. diphtheriae* and *Corynebacterium xerosis* [52], and seems to play an important role in the mobilization of genes associated with the MLS phenotype (resistance to macrolides, lincosamides, and streptogramin) in corynebacteria of clinical importance.

Prophages, common in *C. diphtheriae* complex genomes, contribute to their genomic plasticity [53]. However, the role of these elements in the acquisition of resistance genes by these species is still unclear. Presently, we identify one intact prophage per strain, all from the *Siphoviridae* family, with GC contents exceeding the typical 53.3% for *C. ulcerans* genomes. In strain IHP37393, the *Rhodococcus* phage Jace carries integrase and repressor genes, suggesting that it is a temperate, circularly permuted phage [54]. In IHP103889, the *Gordonia* phage Nyceirae is linked to prophage-mediated viral defense [55], while for IHP106492, the *Corynebacterium* phage Adelaide’s lifestyle remains uncertain (https://phagesdb.org/phages/, accessed on 2 April 2025). Among them, the *Corynebacterium* phage Adelaide has previously been reported in the literature as being associated with antimicrobial resistance genes in a corynebacterial species, *C. striatum* [56].

CRISPR–Cas is an adaptive defense system widely found in many bacteria and archaea that helps them to recognize and destroy invading genetic elements, including viruses, phages, and plasmids [57]. This system is composed of a repeated short array separated by spacers, a leader sequence containing the promoter and located upstream of the CRISPR array, and a set of CRISPR-associated genes (*cas*) which encode Cas proteins with endonuclease activity [58]. In this study, we found the type I-E CRISPR–Cas system in all strains, which has also been detected in other *C. diphtheriae* complex species, including *C. diphtheriae* [59] and *C. rouxii* [13]. However, in our strains, the absence of the *cse*1 gene was observed, which is required for Cas3 to recognize the DNA target site and position itself adjacently to the protospacer-adjacent motif to ensure cleavage [60]. This absence may suggest an incomplete or modified system; however, no conclusive studies addressing the effects of the absence of this gene in species of the *C. diphtheriae* complex have been reported to date. The high diversity of targets for the spacer sequences found in this study, like corynebacteria (*C. ulcerans* and *C. diphtheriae*), *Rhodococcus* phages, and *Streptococcus* phage phiSASD1, highlights a CRISPR–Cas system with the possibility of defense against diverse threats. Moreover, the IHP106492 strain harbored an additional type IU CRISPR–Cas system, containing *cas*2, *cas*1, *cas*3, *csb*2, and *csb*1 genes. This type of CRISPR–Cas system has also been found in *Bifidobacterium* spp. and other components of the intestinal microbiota of humans and animals [61,62,63]. However, even in these species, the type IU CRISPR–Cas system has not yet been well characterized. Additional studies should be performed to characterize this type of CRISPR–Cas system in *C. ulcerans*.

Several virulence factors related to adhesion, invasion, and iron acquisition were identified, supporting the pathogenic potential of this species. All strains harbored three incomplete pilus clusters, *spa*ABC (*srt*A, *spa*C), *spa*DEF (*srt*B, *srt*C), and *spa*GHI (*spa*I), indicating a pilus gene variation possibly linked to altered adherence mechanisms. These clusters are essential for epithelial adherence and colonization [64,65]. The *spa*D gene, encoding a surface-anchored pilus protein, was also present in all strains. Iron acquisition systems, critical for *C. diphtheriae* complex survival, were consistently identified. All strains carried the putative *fag*ABC operon with *fag*D. An in vitro study with the *C. pseudotuberculosis fag*B(C) mutant demonstrated that the *fag* gene expression in the host contributed to virulence when compared to wild-type expression in a goat model of caseous lymphadenitis [66]. Similarly, the *ciu* cluster (*ciu*ABCDE), important for survival under iron limitation, was detected in all isolates [67]. While *C. diphtheriae* possesses both the *hmu*TUV hemin transporter and HtaA-C proteins for hemin uptake [68,69], only the *hmu*TUV cluster was fully present in our *C. ulcerans* strains, suggesting limited hemin utilization, as also observed in *C. rouxii* [14].

Diphtheria toxin production, encoded by *tox*, has been described in *C. diphtheriae*, *C. ulcerans*, and *C. pseudotuberculosis* [70]. In this study, the *tox* gene was absent in all *C. ulcerans* strains, but the *dtx*R gene, encoding the DtxR regulator of DT and siderophore synthesis, oxidative stress response, and other promoters, was detected [71]. Additionally, all strains possessed the *pld* gene, encoding phospholipase D (PLD), a key virulence factor shared with *C. pseudotuberculosis* that is involved in host invasion, persistence, and lesion formation [72,73,74,75]. All strains also carried *emb*C, *mpt*C, and *aft*B genes linked to CdiLAM, contributing to epithelial adherence and cell wall biosynthesis [76,77]. As expected, urease-related genes, including *ure*B and *ure*G, were present in all isolates, distinguishing *C. ulcerans* and *C. pseudotuberculosis* from *C. diphtheriae* [78,79].

As shown in Figure 3, the *rpo*B2 and *rbp*A genes, previously linked to rifampin resistance [44,80], were predicted in all strains. However, these associations are mainly based on gene prediction, while more conclusive studies indicate that rifampin resistance in *Corynebacterium* species is primarily due to point mutations in the housekeeping gene *rpo*B [15]. Similarly, fluoroquinolone resistance is mostly associated with mutations in *gyr*A. In this study, all strains were rifampin-susceptible, and the ciprofloxacin-resistant strain harbored two-point mutations, which we structurally analyzed. Similarly to *gyr*A, the *gyr*B gene encoding the other subunit of DNA gyrase was also detected in all strains.

Fluoroquinolones, such as ciprofloxacin, inhibit bacterial growth by targeting DNA gyrase, preventing resealing of double-stranded DNA breaks, and forming stable enzyme–DNA adducts called cleaved complexes [81]. At higher concentrations, they cause DNA break release, chromosome fragmentation, and cell death [82]. Ciprofloxacin mainly interacts with the α4 helix of GyrA, with additional contact near the C7 group in GyrB. A key residue for resistance is the second position of the α4 helix in GyrA, typically a serine in sensitive strains [83], which stabilizes enzyme–drug binding via a water/magnesium ion bridge [84]. Resistance commonly arises from mutations in this α4 helix region, especially residues 87–94 in *gyr*A of *M. tuberculosis* and *Corynebacterium* spp. [85,86,87].

Previous studies on *Corynebacterium* spp. have shown that amino acid substitutions within the QRDR, particularly the replacement of a polar amino acid with a hydrophobic one at positions 87 and 91, correlate with increased minimum inhibitory concentration (MIC) values and resistance to quinolones, including ciprofloxacin [85,87]. In *C. ulcerans* susceptible to fluoroquinolones, these positions correspond to serine and aspartate at residues 89 and 93, respectively. Both are polar amino acids capable of forming hydrogen bonds with water molecules via their hydroxyl and carboxyl side chains [88], making them crucial for ciprofloxacin binding and overall quinolone efficacy.

Here, we provide significant insights into the antimicrobial resistance and pathogenic potential of *C. ulcerans*, particularly concerning fluoroquinolone resistance. In this study, the mutated *gyr*A sequence (gyr89L-93G) contains leucine and glycine substitutions at positions 89 and 93, respectively. As aliphatic and nonpolar residues, they lack functional groups necessary for hydrogen bonding with water molecules [89], disrupting the interaction between the ciprofloxacin–water/magnesium ion bridge complex and the mutated enzyme. These observations underscore the critical role of this bridge in fluoroquinolone action and may contribute to the development of novel antibacterial agents to combat resistance. Although our in silico analyses provide valuable insights into the structural basis of *gyr*A-mediated fluoroquinolone resistance, further experimental studies, such as gene editing, are necessary to confirm these findings; however, such approaches may be challenging due to the essential and constitutive nature of the *gyr*A gene.

Zoonotically acquired *C. ulcerans* infections are a matter of concern worldwide [23] and highlight the need for surveillance and expansion of knowledge about this pathogen. The present study provided a comprehensive genomic analysis of three *C. ulcerans* strains isolated from domestic animals in Brazil. Although additional studies are still necessary, the data obtained in the present work could contribute to understanding the dissemination and evolution of virulent and antimicrobial resistance strains of this zoonotic pathogen and to the establishment of monitoring, prevention, and treatment measures for *C. ulcerans* infections in humans and animals.

## 4. Materials and Methods

### 4.1. Origin of Bacterial Strains

Three animals from different Brazilian states with signs of discomfort were treated at veterinary clinics. The first one, with an ear injury, was a 14-year-old dog from the State of Paraná. The second one, with a neck abscess, was an 8-year-old cat from the State of Pernambuco. The last one, with an ear injury, was a 3-year-old dog from the State of São Paulo. For analysis, three swabs were collected and sent to Hermes Pardini Institute (Fleury Group), Minas Gerais, Brazil. Bacterial cultures were performed on 5% sheep’s blood agar (Plastlabor^®^, Rio de Janeiro, Brazil) and incubated at 37 °C for 48 h. The semi-automated system VITEK^®^ MS (bioMérieux^®^, Craponne, France) was used to identify isolated strains through MALDI-TOF MS analysis. Bacterial spots of 1 to 3 colonies were placed on the target slide. Then, 1 μL α-cyano-4-hydroxycyanic acid matrix—VITEK MS-CHCA (bioMérieux^®^, Rio de Janeiro, Brazil)—was applied over the samples and air dried until the matrix and samples co-crystallized. The slide was loaded into the VITEK^®^ MS system to acquire protein mass spectra, mainly composed of ribosomal protein. The obtained mass spectra were compared with the MYLA^®^ software version 4.7.1 database (bioMérieux^®^, Craponne, France).

### 4.2. Antimicrobial Susceptibility Testing In Vitro

The antimicrobial susceptibility profiles of all isolates were performed using the disk diffusion method according to the guidelines provided by the BrCAST, a direct translation of the European Committee on Antimicrobial Susceptibility Testing (EUCAST) guidelines officially endorsed by the Brazilian Ministry of Health, valid from April 2024 (https://brcast.org.br/, accessed on 2 April 2025). Bacterial suspensions were prepared in saline to the density of a 0.5 McFarland turbidity standard and seeded on a Mueller–Hinton agar supplemented with 5% defibrinated horse blood and 20 mg/L β-NAD (Plastlabor^®^, Rio de Janeiro, Brazil). Then, the following antimicrobials (Oxoid^®^, São Paulo, Brazil) were placed on the surface of the seeded plates: benzylpenicillin (1 U), ciprofloxacin (5 μg), clindamycin (2 µg), erythromycin (15 µg), linezolid (10 µg), rifampin (5 µg), trimethoprim-sulfamethoxazole (23.75–1.25 µg), and tetracycline (30 μg). Test plates were incubated at 35 ± 1 °C in a 5% CO_2_ atmosphere for 40–44 h. The quality control, which followed BrCAST guidelines, used *Streptococcus pneumoniae* ATCC 49619.

### 4.3. Genome Sequencing, Assembling, and Annotation

Bacterial genomic DNA was extracted using the QIAamp^®^ DNA Blood Mini Kit (QIAGEN^®^, Hilden, Germany) according to the manufacturer’s instructions. Whole-genome sequencing (WGS) was performed using the Illumina^®^ NextSeq 550 platform (Illumina Inc., San Diego, CA, USA). The library was constructed with the DNA Prep Library Preparation Kit (Illumina Inc., San Diego, CA, USA). Sequence quality analysis was performed using FastQC v.0.12.1 (https://github.com/s-andrews/FastQC, accessed on 5 April 2025). All genomes were assembled de novo using Unicycler v.0.5.1 [90], and contigs with less than 200 bp were trimmed. To evaluate the GC content, size, and fragmentation of the genomes, we used QUAST v.5.2.0 [91]. Completeness and contamination levels were estimated using CheckM2 v.1.0.2 [92], and the location of ribosomal RNA genes in genomes was predicted using Barrnap v.0.9 (https://github.com/tseemann/barrnap, accessed on 5 April 2025). Chimerism was checked using GUNC v.1.0.6 [93]. Genomes were annotated using Prokka v.1.14.6 [94] and deposited in GenBank.

### 4.4. Genomic Taxonomy

Taxonomy classification of the strains was performed using TYGS [24] and GTDB-Tk v.2.4.0 [25]. The ANI values among our strains and close reference genomes identified by TYGS were calculated using PyANI v.0.2.12 [95]. The DDH was determined in silico comparing our genomes with the type strains of closely related species—*C. diphtheriae*, NCTC 11397^T^; *C. belfanti*, FRC0043^T^; *C. ramonii*, FRC0011^T^; *C. rouxii*, FRC0190^T^; *C. pseudotuberculosis*, ATCC 19410^T^; *C. silvaticum*, KL0182^T^; and *C. ulcerans*, NCTC 7910^T^—using the Genome-to-Genome Distance Calculator (GGDC) v.3.0 with BLAST+ [96]. The results were based on the recommended formula (2) (identities/HSP length) [97].

### 4.5. Multilocus Sequence Typing (MLST) Characterization and Phylogenetic Analysis

Considering the seven housekeeping genes, *atp*A, *dna*E, *dna*K, *fus*A, *leu*A, *odh*A, and *rpo*B, obtained from the whole genome, sequence type (ST) was determined in silico using the Institute Pauster MLST database (https://bigsdb.pasteur.fr/diphtheria/, accessed on 28 April 2025). The sequences of these seven housekeeping genes were also deposited in this database, which provides access to genotyping data for *C. diphtheriae* complex isolates worldwide.

We used the Genbank database from the National Center of Biotechnology Information (NCBI—https://www.ncbi.nlm.nih.gov, accessed on 30 April 2025) to retrieve some genomic sequences of *C. ulcerans* that we consider important and related to our study. Before downloading the genomes in nucleotide FASTA format, we checked their completeness and contamination levels using CheckM2 v.1.0.2 [92]. The genome of *C. silvaticum* KL0182^T^ was added as an outgroup. For multiple-sequence alignment of the core genome, we used PPanGGOLiN v.2.2.0 [98] with MAFFT with default options to perform the alignment [99]. SNP-sites v.2.5.1 [100] was chosen to extract single-nucleotide polymorphisms (SNPs). The evolutionary model and phylogenetic inference were estimated by IQ-TREE2 v.2.0.7, with the maximum likelihood method. The support values were calculated using 1000 bootstrap replications. The tree was visualized using iTOL v.6 [101].

### 4.6. Prediction of Mobile Genetic Elements and CRISPR–Cas Systems

PlasmidFinder v.2.1.6 was used for in silico detection of plasmids [26], and IntegronFinder v.2.0 was used for identifying and analyzing integrons across the genomes [27]. The ISs were identified using ISEScan v.1.7.2.3 [28]. Prophage sequences were identified and annotated with PHASTEST [29]. Intact prophage regions and their predicted phage genes were visualized using Circular Genome View Server in Proksee [102].

CRISPRCasFinder v.4.2.30 was used to analyze the presence of CRISPR–Cas systems [30]. We only included CRISPR arrays with evidence levels equal to 3 or 4 [30], and the type of CRISPR–Cas cassette was determined according to the previously described nomenclature and classification [103]. Spacer sequences were analyzed for their identity in the CRISPRTarget database [31]. Spacer hits were selected from the CRISPRTarget with a lower cut-off identity cover (IC) score of 0.80 [104].

### 4.7. Identification of Genes Encoding Antimicrobial Resistance and Virulence Factors

VFanalyzer was implemented to screen potential virulence factors using the VFDB (Virulence Factor Database) database [105]. Furthermore, PanViTa v.1.1.3 [106] was used to search for antimicrobial resistance genes and virulence genes using the CARD (Comprehensive Antibiotic Resistance Database) and VFDB databases, respectively. For more accurate results, we also used the BlastKOALA server [107,108]. Circular genome map comparisons were created with *C. ulcerans* NCTC 7910^T^ as a reference using BRIG (Blast Ring Image Generator) software v.0.95 [108] to show the positions of virulence factors and antimicrobial resistance genes.

### 4.8. Mutation Analysis

For the identification of mutations in the QRDR in our strains, we used the susceptible DNA gyrase sequence obtained from *C. ulcerans* strain 809 (GenBank accession number: GCA_000215645.1) [32]. This sequence was aligned against the IHP37393 strain (which is resistant to ciprofloxacin) and with the IHP103889 and IHP106492 strains (which is susceptible to increased exposure to ciprofloxacin). The alignment was performed using MUSCLE in MEGA software v.11.0.13 with default parameters [97] and analyzed using Jalview v.2.4.11.1 [109].

The 3D structure of the IHP37393 DNA gyrase protein variant (named gyr89L-93G here) was predicted using the Swiss-Model server [110]. The model was built using the DNA gyrase complex structure available in the Protein Data Bank (PDB; https://doi.org/10.2210/pdb5bta/pdb), which was determined by X-ray crystallography at a resolution of 2.55 Å. This reference structure shares 78.11% sequence identity with our protein and covers 48% of its length. After building the model, we adjusted it to represent the correct protonation state at pH 7.0 using the CHARMM-GUI web server [111]. We then optimized the structure by reducing any unfavorable atomic interactions through 5000 steps of energy minimization, using the steepest descent method in GROMACS 2023 [112]. Finally, the quality of the model was evaluated with the MolProbity web [113].

Molecular docking was performed using DockThor [114] with the *M. tuberculosis* DNA gyrase complexed with ciprofloxacin (redocking) (https://doi.org/10.2210/pdb5btc/pdb), which shares 76.0% sequence identity with gyr89L-93G. During the docking process, we included the magnesium ion, which is described as involved in protein–ligand interaction, along with its associated water molecules [83]. Similarly, molecular docking was performed with ciprofloxacin and the gyr89L-93G structure, also accounting for the magnesium ions and their associated water molecules.

Molecular dynamics simulations were carried out in GROMACS 2023 using the CHARMM36 force field [115]. The protein–ligand system was placed in a virtual box filled with TIP3P water molecules and neutralized by adding counterions. To negate any steric clashes or high-energy interactions, the system was first energy-minimized using the steepest descent method until the maximum force dropped below 1000 kJ/mol/nm. After minimization, the system was gradually brought to a temperature of 310 K (approximately human body temperature) while keeping the protein fixed, allowing the solvent and ions to settle around it. The system was then equilibrated at 310 K before running a 100-nanosecond simulation under near-physiological conditions: pH 7.0, 1 atm pressure, and 25 °C. During the simulation, we monitored the protein’s structural stability using the root mean square deviation (RMSD), its flexibility using the root mean square fluctuation (RMSF), and its compactness using the radius of gyration (Rg). GROMACS routines were used to calculate the root mean square deviation (RMSD), the root mean square fluctuation (RMSF) for the protein and ligand backbone, and the radius of gyration (Rg) of the protein.

Ciprofloxacin parameters for the simulation were generated with the CGenFF web server [116], following established GROMACS protein–ligand simulation protocols [117]. All simulation plots were generated using Xmgrace (Grace-5.1.25). The 2D interaction maps were produced with LigPlot+ v.2.2.9 [118], while 3D structural representations of the protein–ligand complexes and docking analyses were visualized using PyMOL v.2.5.0 (the PyMOL Molecular Graphics System, v.1.2r3pre, Schrödinger, New York, NY, USA).

## Figures and Tables

**Table 1:** Genomic features of *Corynebacterium ulcerans* IHP37393, IHP103889, and IHP106492 strains.

Feature	Strain		
	IHP37393	IHP103889	IHP106492
Accession number	JBGNWQ000000000	JBGNWN000000000	JBGNWM000000000
Platform	Illumina^®^ NextSeq 550	Illumina^®^ NextSeq 550	Illumina^®^ NextSeq 550
Completeness (%)	99.99	99.99	99.99
Contamination (%)	0.74	0.29	0.60
Coverage	251	293	261
Chimerism	No	No	No
Total length (bp)	2,489,063	2,496,757	2,521,744
GC (%)	53.3	53.3	53.3
Contigs	6	6	5
N50	815,040	808,602	828,787
L50	2	2	2
CDS	2242	2252	2293
rRNAs	3	3	3
tRNAs	51	51	51

## Data Availability

All data generated or analyzed during this study are included in this published article. The whole-genome sequences of the *Corynebacterium ulcerans* IHP37393, IHP103889, and IHP106492 strains were uploaded to the NCBI with the accession numbers JBGNWQ000000000, JBGNWN000000000, and JBGNWM000000000, respectively.

## Supplementary Materials

The following supporting information can be downloaded at https://www.mdpi.com/article/10.3390/antibiotics14080843/s1: Figure S1: Heatmap representing the ANI percentage nucleotide identity of all matching regions between IHP37393, IHP103889, and IHP106492 isolates and the closest related type strains; Table S1: DDH in silico results obtained using GGDC v.3.0 for *C. ulcerans* strains compared to the closest related type strains; Figure S2: Linear genome map visualized using Circular Genome View Server in Proksee, showing the location of the cas genes and some CRISPR arrays; Table S2: Number of predicted IS families in each *C. ulcerans* strain using ISEScan v.1.7.2.3; Figure S3: Multiple sequence alignment of *C. ulcerans* DNA gyrase; Table S3: Hits found for spacer sequences in the CRISPRTarget databases; Figure S4: Quality assessment of the gyr89L-93G model.
