# Supplementary material for: Quinolone Resistance and Zoonotic Potential of Corynebacterium ulcerans from Domestic Animals in Brazil
Source: Antibiotics (Basel). 2025 Aug 20;14(8):843. doi: 10.3390/antibiotics14080843 (PMC12383154; doi:10.3390/antibiotics14080843)
Supplement: Supplementary file 1 [file antibiotics-14-00843-s001.zip › antibiotics-3756259-supplementary.pdf]

antibiotics

Supplementary Material

Quinolone Resistance and Zoonotic Potential of *Corynebacterium ulcerans* from Domestic Animals in Brazil

Fernanda Diniz Prates [1,2†*], Max Roberto Batista Araújo [1,2†], Jailan da Silva Sousa[2], Lincoln de Oliveira Sant'Anna[3], Tayná do Carmo Sant'Anna Cardoso[3], Amanda Couto Calazans Silva[3,] Siomar de Castro Soares[4], Bruno Silva Andrade[5,6], Louisy Sanches dos Santos[3], Vasco Ariston de Carvalho Azevedo[2*]

[1] Operational Technical Nucleus, Microbiology, Hermes Pardini Institute, Vespasiano, Minas Gerais, Brazil
[2] Institute of Biological Sciences, Federal University of Minas Gerais, Belo Horizonte, Minas Gerais, Brazil
[3] Laboratory of Diphtheria and Corynebacteria of Clinical Relevance, Department of Microbiology, Immunology and Parasitology, Rio de Janeiro State University, Rio de Janeiro, Rio de Janeiro, Brazil
[4] Institute of Biological and Natural Sciences, Federal University of Triângulo Mineiro, Uberaba, Minas Gerais, Brazil
[5] Laboratory of Bioinformatics and Computational Chemistry, Department of Biological Sciences, State University of Southwest of Bahia, Jequié, Bahia, Brazil
[6] INRAE - France's National Research Institute for Agriculture, Food and Environment, STLO, Rennes, France
* Correspondence: fernandaprates3@hotmail.com; vascoariston@gmail.com
† These authors contributed equally to this work.

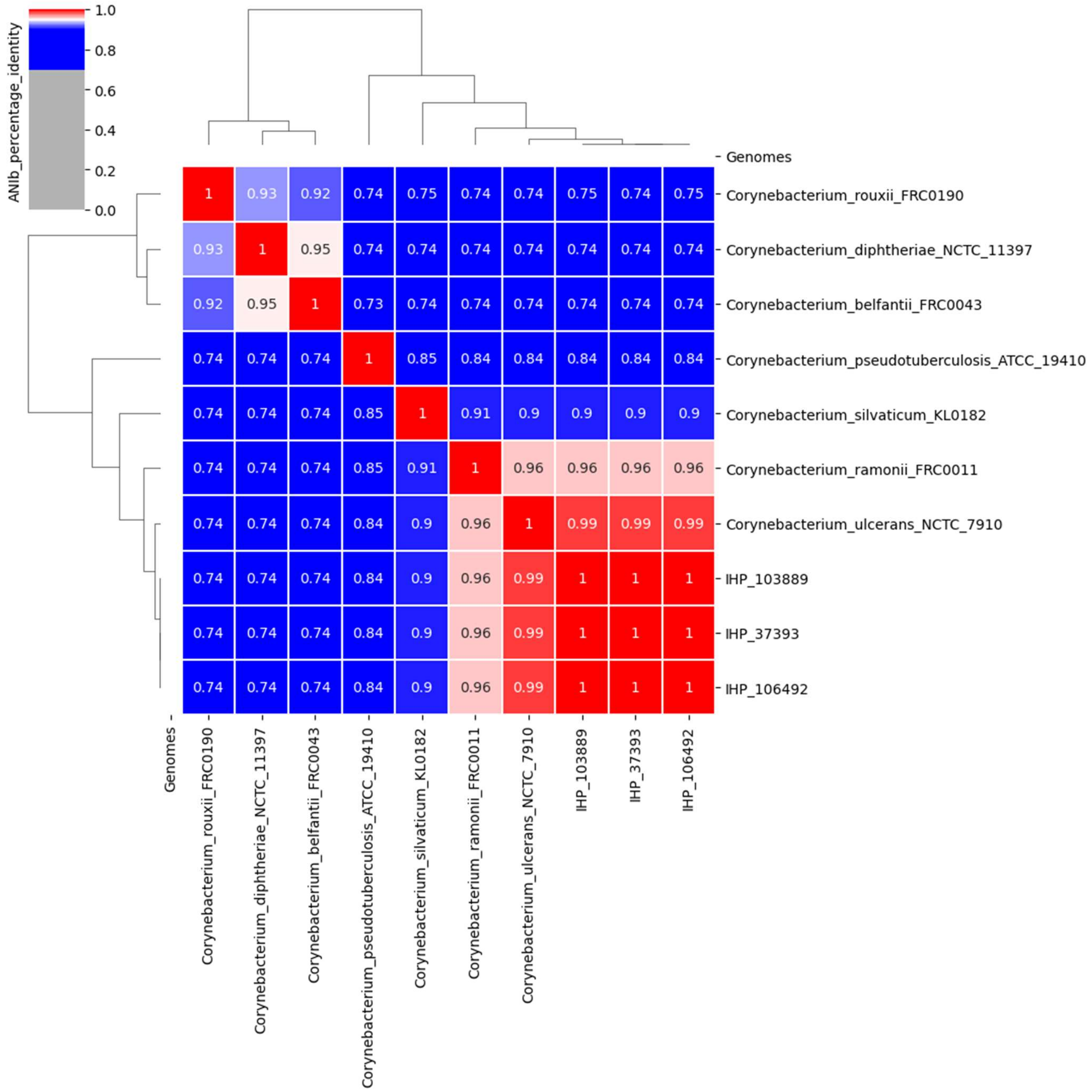

Figure S1. Heatmap representing the ANI percentage nucleotide identity of all matching regions between IHP37393, IHP103889, IHP106492 isolates and the closest related type strains using PyANI v.0.2.12. The default scheme colors cells with identity > 0.95 as red, and those with < 0.95 as blue.

antibiotics MDPI

Table S1. DDH in silico results obtained by GGDC v.3.0 for *Corynebacterium ulcerans* strains compared to the closest related type strains.

Strain	*C. ulcerans* NCTC 7910^T	*C. ramonii* FRC0011^T	*C. silvaticum* KL0182^T	*C. pseudotuberculosis* ATCC 19410^T	*C. diphtheriae* NCTC 11397^T	*C. rouxii* FRC0190^T	*C. belfantii* FRC0043^T
IHP37393	88.5%	66.4%	41.1%	27.6%	23.2%	22.6%	22.2%
IHP103889	88.1%	66.3%	40.9%	27.6%	23.2%	22.8%	22.1%
IHP106492	88.4%	66.4%	41.1%	27.6%	23.2%	22.5%	22.2%

Table S2. Number of predicted IS family in each one of *Corynebacterium ulcerans* strain using ISEScan v.1.7.2.3.

Strain	Family	Cluster	IS Begin	IS End	IS Length	Copy number	Genome coordinates of the predicted Tpase ORF		Strand where the Tpase is	Length of predicted Tpase OR
							Start	End		
IHP37393	IS110	IS110_139	676947	677991	1045	1	676918	677673	-	756
	IS256	IS256_174	697310	698720	1411	1	697475	698493	+	1019
	IS21	IS21_259	732527	734720	2194	1	732652	734478	-	1827
	IS110	IS110_139	243303	244616	1314	1	243646	244453	-	808
IHP103889	IS110	IS110_139	258636	259949	1314	1	258979	259786	-	808
	IS110	IS110_139	630140	631184	1045	1	630111	630866	-	756
	IS256	IS256_174	650503	651913	1411	1	650668	651686	+	1019
	IS21	IS21_259	686984	689177	2194	1	687109	688935	-	1827
	IS110	IS110_139	301428	302741	1314	1	301771	302578	-	808
	IS21	IS21_259	119426	121619	2194	1	119668	121494	+	1827
	IS256	IS256_174	156629	158039	1411	1	156856	157874	-	1019
IHP106492	IS110	IS110_139	177358	178402	1045	1	177676	178431	+	756

Table S3. Hits found to spacer sequences in the CRISPRTarget databases.

Strain	Cas-Type	*cas* genes	CRISPRTarget				
			Number of spacers	Evidence Level	Spacer	IC score	Identity
IHP37393	I-E	*cas5, cas7, cse2, cas6, cas3, cas1, cas2*	10	4	7	1.0	*C. ulcerans* 210931
					9	0.94	*C. ulcerans* FRC58
			14	4	13	0.88	*C. ulcerans* FRC58
					14	1.0	*C. ulcerans* 0102
			31	4	1	0.88	*C. ulcerans FRC58*
					7	1.0	*C. ulcerans* 0102, 131001, 210932
					7	0.94	*C. ulcerans* FRC58
					8	0.94	*C. ulcerans* 210931
					8	0.88	*C. ulcerans* BR-AD22
					8	0.81	*C. ulcerans* 0102, 131001, 210932
					9	0.94	*C. ulcerans* 0102, BR-AD22
					13	0.81	*C. ulcerans BR-AD22*
					13	0.81	*C. diphtheriae* 31A
					13	0.81	Phage *Rhodococcus* RRH1
					14	1.0	*C. ulcerans* 0102, BR-AD22
					17	1.0	*C. ulcerans* BR-AD22
					17	1.0	Phage *Rhodococcus* REQ3
					17	0.94	*C. ulcerans* 0102
					21	1.0	*C. ulcerans* 0102, BR-AD22
					21	1.0	Phage *Rhodococcus* REQ3
					23	1.0	*C. ulcerans* FRC58
					24	0.88	*C. ulcerans* FRC58
					25	0.81	*C. diphtheriae B-D-16-78*

				30	1.0	*C. ulcerans* BR-AD22
IHP103889	I-E	*cas5, cas7, cse2, cas6, cas3, cas1, cas2*	48	4	1	0.88	*C. ulcerans* FRC58
					8	1.0	*C. ulcerans* 0102, 131001, 210932
					8	0.94	*C. ulcerans* FRC58
					9	0.94	*C. ulcerans* 210931
					9	0.88	*C. ulcerans* BR-AD22
					9	0.81	*C. ulcerans* 0102, 131001, 210932
					10	0.94	*C. ulcerans* 0102, BR-AD22
					14	0.81	*C. diphtheriae* 31A
					14	0.81	*C. ulcerans* BR-AD22
					14	0.81	Phage *Rhodococcus* RRH1
					15	1.0	*C. ulcerans* 0102, BR-AD22
					17	0.94	*C. ulcerans* BR-AD22
					17	0.94	Phage *Streptococcus* phiSASD1
					34	1.0	*C. ulcerans* BR-AD22
					34	1.0	Phage *Rhodococcus* REQ3
					34	0.94	*C. ulcerans* 0102
					38	1.0	*C. ulcerans* 0102, BR-AD22
					38	1.0	Phage *Rhodococcus* REQ3
					40	1.0	*C. ulcerans FRC58*
					41	0.88	*C. ulcerans FRC58*
					42	0.81	*C. diphtheriae B-D-16-78*
					47	1.0	*C. ulcerans BR-AD22*
			10	4	7	1.0	*C. ulcerans* 210931
					9	0.94	*C. ulcerans* FRC58
					4	0.88	*C. ulcerans BR-AD22*
					4	0.88	Phage *Rhodococcus* REQ3

			24	4	4	0.81	*C. ulcerans* 0102
					7	0.88	*C. ulcerans* FRC58
					7	0.81	*C. ulcerans* 0102
					8	1.0	*C. ulcerans* 0102
					8	0.94	*C. ulcerans* BR-AD22
					13	1.0	Phage *Rhodococcus* REQ3
					13	1.0	*C. ulcerans* 0102, BR-AD22
					14	1.0	*C. ulcerans* 0102, BR-AD22
					15	0.81	Phage *Rhodococcus* REQ3
					15	0.81	*C. ulcerans* BR-AD22
					17	1.0	*C. ulcerans* FRC58
IHP106492	I-E	*cas5, cas7, cse2, cas6, cas3, cas1, cas2*	22	4	1	0.88	*C. ulcerans* FRC58
					6	1.0	*C. ulcerans* 0102, 131001, 210932
					6	0.94	*C. ulcerans* FRC58
					7	0.81	*C. diphtheriae* 31A
					7	0.81	Phage *Rhodococcus* RRH1
					7	0.81	*C. ulcerans* BR-AD22
					8	1.0	*C. ulcerans* 0102, BR-AD22
					12	1.0	Phage *Rhodococcus* REQ3
					12	1.0	*C. ulcerans* 0102, BR-AD22
					14	1.0	*C. ulcerans* FRC58
					15	0.88	*C. ulcerans FRC58*
					16	0.81	*C. diphtheriae* B-D-16-78
	IU	*cas2, cas1, cas3, csb2, csb1*	23	4	4	0.88	*C. ulcerans* 0102, BR-AD22
					7	1.0	*C. ulcerans FRC58*
					9	0.81	Phage *Rhodococcus* REQ3
					9	0.81	*C. ulcerans* BR-AD22

			10	1.0	*C. ulcerans* 0102, BR-AD22
			11	1.0	Phage *Rhodococcus* REQ3
			11	1.0	*C. ulcerans* 0102, BR-AD22
			14	0.81	*C. ulcerans* FRC58
			18	1.0	*C. ulcerans* 0102
			18	0.94	*C. ulcerans* BR-AD22
	10	4	2	0.91	*C. ulcerans* FRC58
			5	1.0	*C. ulcerans* 210931
			5	0.81	*C. ulcerans* 0102

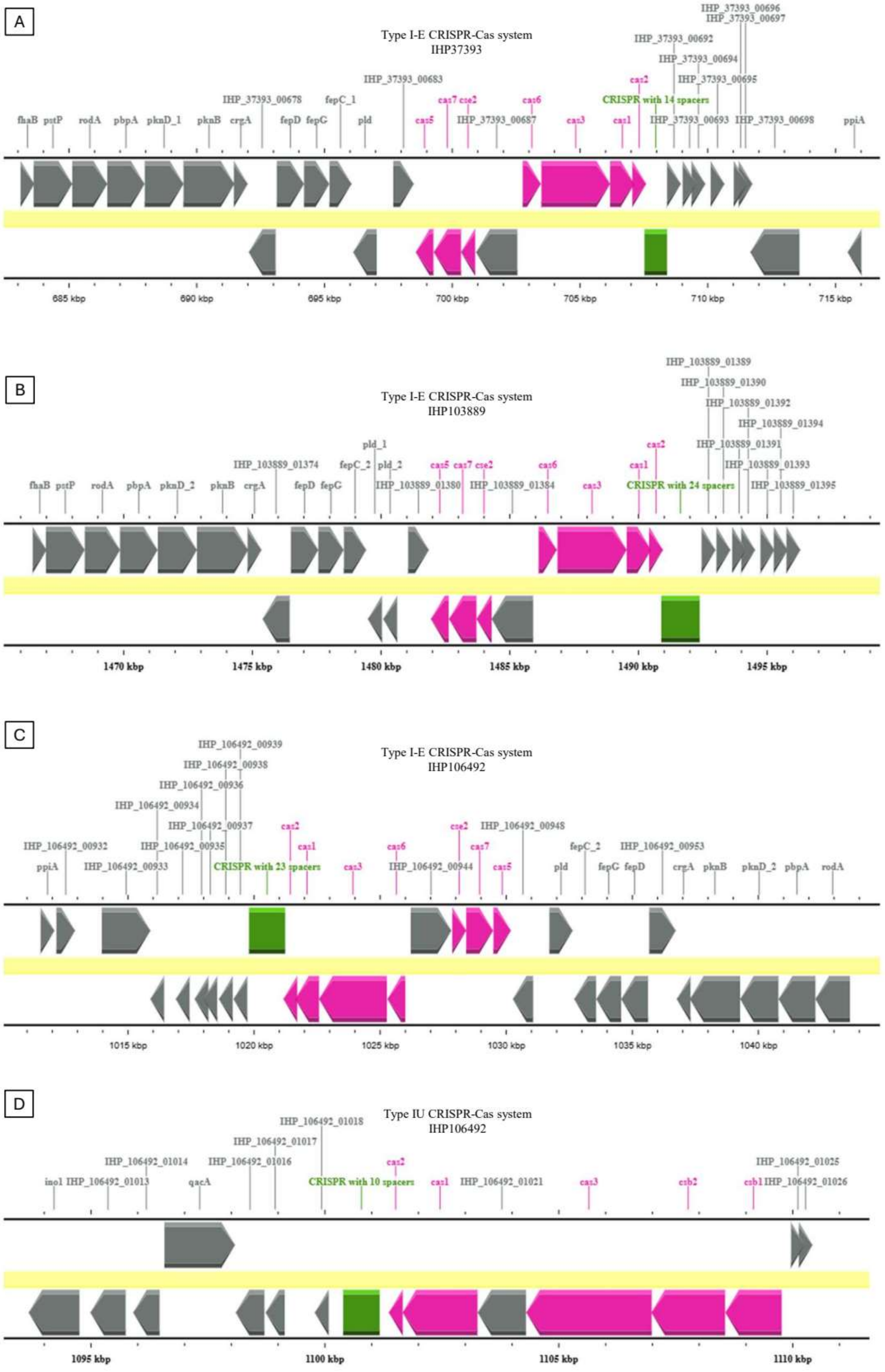

Figure S2. Linear genome map visualized using Circular Genome View Server in Proksee showing the location of the *cas* genes and some CRISPR arrays in the IHP37393 (A), IHP103889 (B) and IHP106492 (C, D) strains.

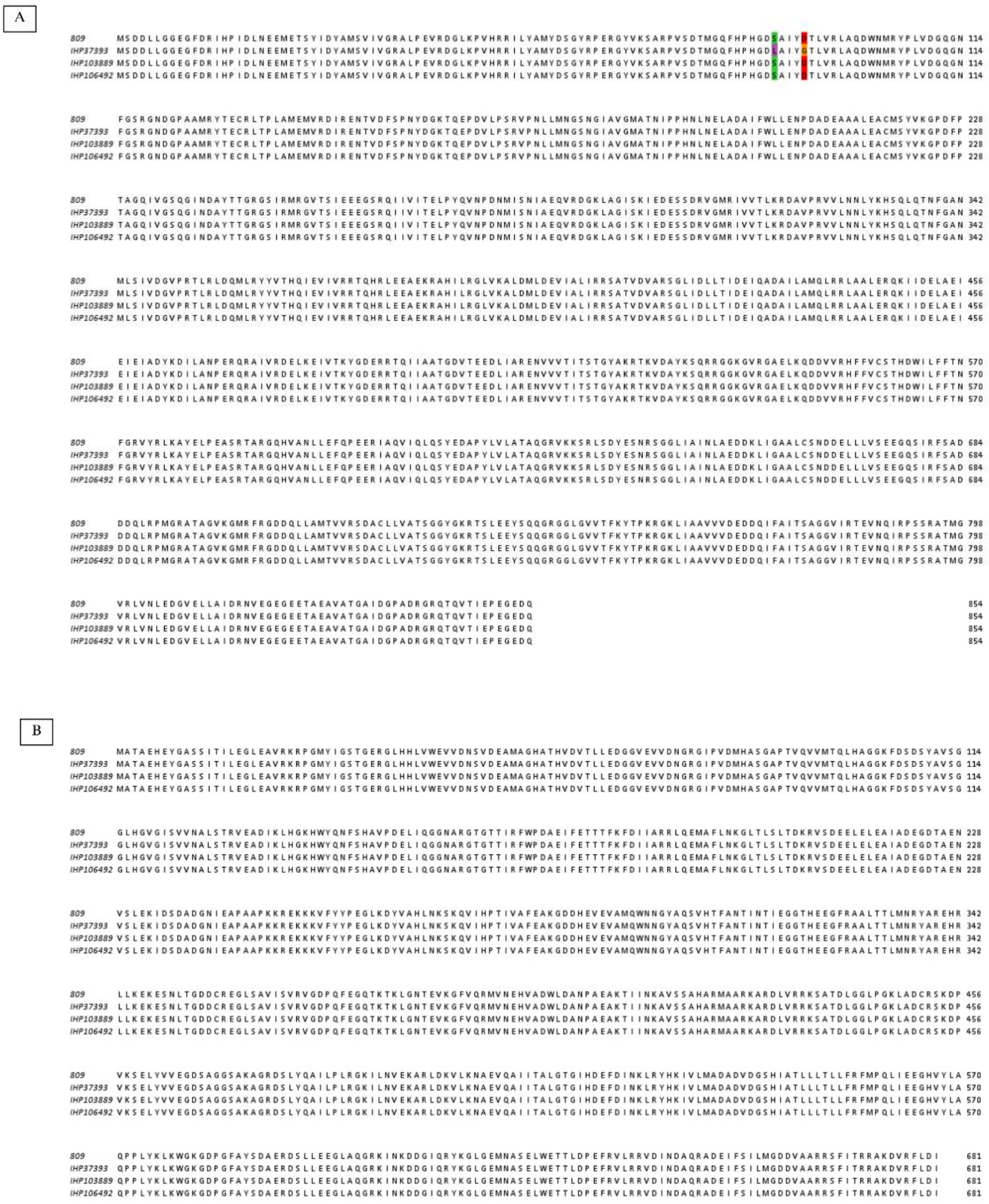

Figure S3. Multiple sequence alignment of *Corynebacterium ulcerans* DNA gyrase. (A) GyrA subunit, highlighting mutations in the ciprofloxacin resistant IHP37393 strain. (B) No mutations were identified in the GyrB subunit of the resistant strain in comparison to susceptible ones.

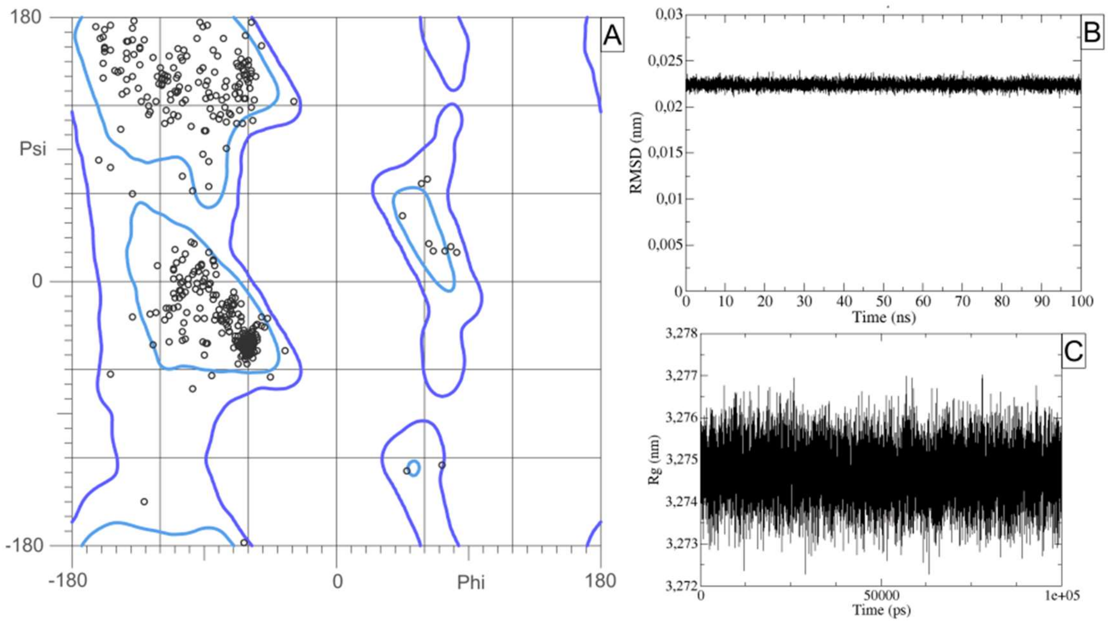

Figure S4. Quality assessment of the gyr89L-93G model. (A) Ramachandran plot showing amino acid residues in favorable and allowed regions. (B, C) RMSD and radius of gyration (Rg) plots, illustrating protein stability during the simulation.
